# Context-aware lightweight remote-sensing image super-resolution network

**DOI:** 10.3389/fnbot.2023.1220166

**Published:** 2023-06-23

**Authors:** Guangwen Peng, Minghong Xie, Liuyang Fang

**Affiliations:** ^1^Faculty of Information Engineering and Automation, Kunming University of Science and Technology, Kunming, China; ^2^Yunnan Key Laboratory of Digital Communications, Kunming, China

**Keywords:** convolutional neural network, transformer, remote-sensing image super-resolution, lightweight network, context-aware

## Abstract

In recent years, remote-sensing image super-resolution (RSISR) methods based on convolutional neural networks (CNNs) have achieved significant progress. However, the limited receptive field of the convolutional kernel in CNNs hinders the network's ability to effectively capture long-range features in images, thus limiting further improvements in model performance. Additionally, the deployment of existing RSISR models to terminal devices is challenging due to their high computational complexity and large number of parameters. To address these issues, we propose a Context-Aware Lightweight Super-Resolution Network (CALSRN) for remote-sensing images. The proposed network primarily consists of Context-Aware Transformer Blocks (CATBs), which incorporate a Local Context Extraction Branch (LCEB) and a Global Context Extraction Branch (GCEB) to explore both local and global image features. Furthermore, a Dynamic Weight Generation Branch (DWGB) is designed to generate aggregation weights for global and local features, enabling dynamic adjustment of the aggregation process. Specifically, the GCEB employs a Swin Transformer-based structure to obtain global information, while the LCEB utilizes a CNN-based cross-attention mechanism to extract local information. Ultimately, global and local features are aggregated using the weights acquired from the DWGB, capturing the global and local dependencies of the image and enhancing the quality of super-resolution reconstruction. The experimental results demonstrate that the proposed method is capable of reconstructing high-quality images with fewer parameters and less computational complexity compared with existing methods.

## 1. Introduction

The aim of single image super-resolution (SISR) is to reconstruct a high-resolution image from its associated low-resolution version. As a low-level visual task within the realm of computer vision, SISR algorithms serve to recover lost texture details in low-resolution images, thereby providing enhanced clarity for higher-level visual tasks, such as person re-identification (Li et al., 2022b, 2023a; Li S. et al., 2022; Zhang et al., 2022), medical imaging (Georgescu et al., 2023), image dehazing/defogging (Zheng et al., 2020; Zhu et al., 2021c), low-resolution image fusion (Li et al., 2016, 2021; Xiao et al., 2022), and remote sensing (Chen L. et al., 2021; Jia et al., 2023). In the field of remote sensing, high-resolution remote-sensing images can be used to obtain more detailed information about the detected area. The most direct method to obtain high-resolution remote-sensing images is to improve the precision of CMOS or charge-coupled device sensors. However, this approach entails substantial costs (Xu et al., 2021). On the contrary, SISR technology can economically and conveniently improve the resolution of remote-sensing images.

In recent years, the advancement of deep learning has led to the proposal of numerous convolutional neural network (CNN)-based SISR methods, which have demonstrated remarkable performance. Dong et al. (2014) were the pioneers in applying CNNs to super-resolution (SR) tasks, introducing the Super-Resolution Convolutional Neural Network (SRCNN). SRCNN employs bicubic interpolation to enlarge the input low-resolution image to the target size and utilizes a three-layer convolutional network for nonlinear mapping to obtain a high-resolution image. This approach outperforms traditional super-resolution reconstruction methods. However, SRCNN suffers from high computational complexity and slow inference speed. To overcome these limitations, Dong et al. (2016) proposed the Fast Super-Resolution Convolutional Neural Network (FSRCNN), building upon SRCNN to directly extract features from low-resolution images, thus accelerating computation. Kim et al. introduced a Deeply-Recursive Convolutional Network (DRCN; Kim et al., 2016b) and a Very Deep Convolutional Network for Image Super-Resolution (VDSR; Kim et al., 2016a), both of which employ deeper convolutional layers and have achieved impressive results in SR tasks. This supports the notion that deeper CNNs can enhance model performance. Lim et al. (2017) proposed Enhanced Deep Residual Networks for Single Image Super-Resolution (EDSR), incorporating a deeper network and residual structure, further emphasizing that deeper networks yield superior super-resolution performance. Although high-resolution images can be obtained using the above SISR approaches, computational costs and memory consumption need to be considered when deploying the models on mobile devices, especially in the field of remote sensing (Qi et al., 2022; Liu Y. et al., 2023; Liu Z. et al., 2023; Wang et al., 2023). Wang et al. (2022) proposed a lightweight feature enhancement network (FeNet) for remote-sensing image super-resolution, which aims to achieve high-quality image reconstruction by effectively extracting and enhancing image features. FeNet can maintain high reconstruction quality while reducing computational complexity and memory consumption. Nonetheless, due to the limited receptive field of the convolution kernel, CNN-based super-resolution models can only acquire local image information during convolution operations, which restricts their performance. Consequently, super-resolution networks need to extract both global and local information from images to achieve further improvements in performance.

Transformer (Vaswani et al., 2017) differs significantly from CNNs and is capable of capturing global information in images through its self-attention mechanism. Consequently, Liang et al. (2021) designed an image restoration network called SwinIR, which combines CNNs and Transformers. This network effectively models long-range dependencies in images, facilitating the restoration of global image information. However, SwinIR only relies on CNNs to extract shallow features, neglecting to fully exploit the CNN's potential to capture local information in intermediate layers. This results in the model's limited ability to acquire local information. Tu et al. (2022) proposed a generative adversarial network (GAN) called SWCGAN, which aims to address the limitations of convolutional layers in modeling long-range dependencies and uses a combination of Swin Transformer and convolutional layers to generate high-resolution remote-sensing images. To further investigate the aggregation of local and global information, Chen et al. (2022) and Gao et al. (2022b) proposed the image super-resolution networks HAT and LBNet, respectively. HAT employs a hybrid attention mechanism, combining channel attention and self-attention to activate more pixels, thereby enhancing the quality of super-resolution reconstruction images. Nevertheless, the hybrid attention mechanism leads to a substantial increase in the model's number of parameters and computational complexity. LBNet fuses symmetric CNNs with recursive Transformers to offer a high-performance, efficient solution for SISR tasks. However, LBNet directly cascades the CNN and recursive Transformer, overlooking the dynamic interaction between global and local information during the feature extraction process. Thus, further research is warranted to effectively harness the local feature extraction capabilities of CNNs and the global feature extraction capacities of Transformers to improve the performance of SISR models.

**Figure 1:**
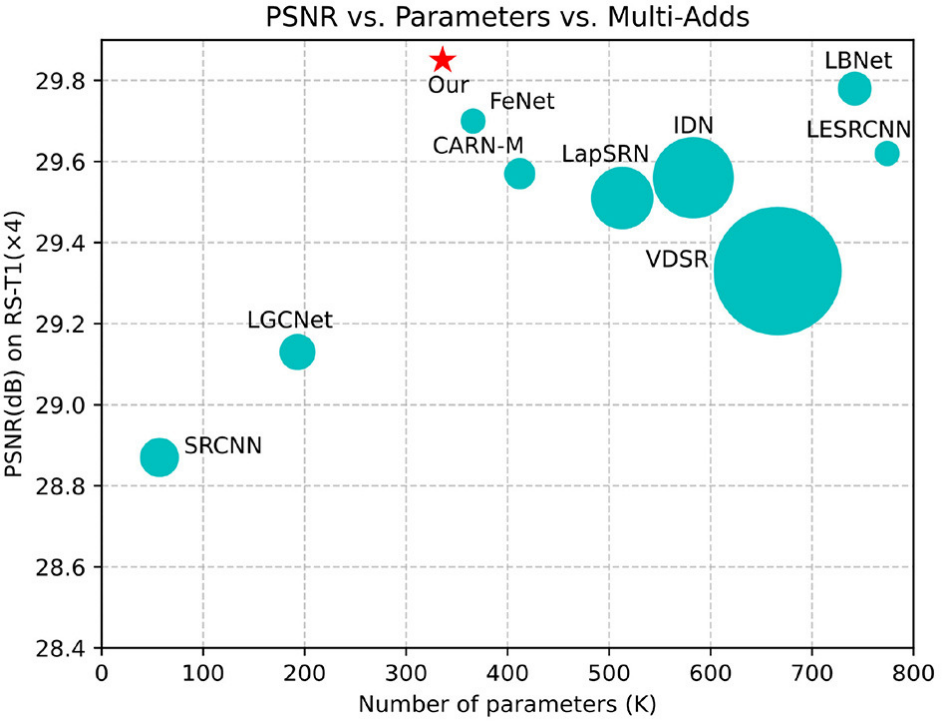
Comparison of SISR models (×4) in terms of accuracy, network parameters, and Multi-Adds from the RS-T1 dataset. The area of each circle denotes the number of Multi-Adds. The proposed model achieves comparable performance with fewer parameters and lower Multi-Adds. The star symbol represents the model proposed by us.

To address the above issues, we propose a context-aware lightweight super-resolution network (CALSRN) for remote-sensing images. This novel network is capable of extracting both local and global features from images and dynamically adjusting their fusion weights, thereby better representing image information and enhancing reconstruction quality. Furthermore, the proposed model has only about 320 K parameters, making it lighter than existing state-of-the-art lightweight super-resolution reconstruction networks while maintaining superior performance, as shown in Figure 1. This lower number of parameters results in lower computational complexity of the model. Overall, the proposed network achieves a good balance between performance and model complexity. In summary, our main contributions are as follows.

1. We propose a lightweight remote-sensing image SR network consisting of ~320 K parameters. In comparison to other state-of-the-art lightweight SR networks, the proposed network demonstrates the ability to reconstruct higher-quality images with a reduced number of parameters and lower computational complexity, thereby facilitating easier deployment on terminal devices.
2. We introduce a context-aware Transformer block (CATB) that is designed to not only capture local details but also concentrate on extracting global features. Simultaneously, dynamic adjustment branches are incorporated to adaptively learn the fusion weights between local and global features, resulting in a more effective feature representation and an enhanced quality of SR reconstruction images.
3. The experimental results demonstrate that the super-resolution reconstruction images generated by the proposed method exhibit substantial structural and textural details. Compared with other lightweight SISR networks, the proposed method achieves the optimum in terms of visual quality and performance evaluation.

## 2. Related works

### 2.1. CNN-based SISR

In recent years, deep learning has been widely used in SISR in view of its excellent performance in image processing (Zhu et al., 2021a; Li et al., 2022a, 2023b; Tang et al., 2023) and recognition (Li et al., 2020; Zhu et al., 2021b; Yan et al., 2022). Dong et al. (2014) first applied CNN to image super-resolution and proposed SRCNN, which outperforms the conventional SISR methods. To solve the problem of slow inference speed of SRCNN, Dong et al. (2016) improved SRCNN and proposed FSRCNN. To overcome the limitation of the limited receptive field of the convolutional kernel, Lei et al. (2017) proposed the local global combined network (LGCNet), which extracts local and global features in low-resolution images by local and global networks, respectively, and combines these two features together for super-resolution image reconstruction. Lai et al. (2017) Laplacian pyramid super-resolution networks (LapSRN), which combined Laplacian pyramid with deep learning to achieve multi-level super-resolution reconstruction. Since simply stacking convolutional layers may lead to gradient explosion, Kim et al. (2016a) proposed VDSR, which alleviates the gradient explosion problem by residual learning. From the perspective of reducing the model parameters and computational complexity, DRRN (Tai et al., 2017a), MemNet (Tai et al., 2017b), and LESRCNN (Tian et al., 2020) use a recursive approach to increase the sharing of model parameters. These methods have achieved good performance for remote-sensing image super-resolution. However, their inference speed is limited by the fact that recurrent networks require deeper CNNs for information compensation. Hui et al. (2018) proposed information distillation network (IDN), which extracts detail and structural information through a knowledge distillation strategy to obtain better performance while reducing the model parameters. Lan et al. (2021) proposed a lightweight SISR model named MADNet, which effectively combines multi-scale residuals and attention mechanisms to enhance image feature representation. To effectively extract and fuse features from different levels, Lan et al. (2021) proposed a feature distillation and interaction weighting strategy to improve the super-resolution image quality. However, the number of parameters of the above models is still large. In order to address the limitations of memory consumption and computational burden in remote-sensing image super-resolution applications, Wang et al. (2022) proposed a lightweight feature enhancement network (FeNet) for accurate remote-sensing image super-resolution reconstruction. FeNet uses lightweight lattice blocks (LLB) and feature enhancement blocks (FEB) to extract and fuse features with different texture richness. FeNet has a smaller number of model parameters and faster inference speed, but its ability to capture global information is limited due to the constraints of convolutional kernel receptive field. In general, lightweight CNN-based SISR networks have difficulty in capturing global information of images, while CNN-based models with a larger number of parameters are challenging to deploy directly on terminal devices. Consequently, we design a lightweight SISR network capable of capturing both local and global image features.

### 2.2. Transformer-based SISR

In recent years, Transformer (Vaswani et al., 2017) has been applied to low-level computer vision tasks with good results due to its global feature capture capability. Chen H. et al. (2021) proposed a pre-trained image processing Transformer for image recovery. Liang et al. (2021) proposed SwinIR network by migrating the Swin Transformer (Liu et al., 2021) directly to the image recovery task with good results. However, the dual layer structures in the Swin Transformer block all use multi-head self-attention, which makes the SwinIR too complex. Lu et al. (2021) proposed an effective Transformer for SISR, which reduces GPU memory consumption through lightweight Transformers and feature separation strategies. Chen et al. (2022) proposed a SISR Transformer named HAT. HAT employs a hybrid attention combining channel attention and self-attention, while introducing an overlapping cross-attention module to better aggregate information across windows, achieving good super-resolution performance. However, the number of parameters in HAT is too large. Chen et al. (2022) proposed a lightweight super-resolution network LBNet, which combines CNN and Transformer. In LBNet, the symmetric CNN structure facilitates local feature extraction, and the recursive Transformer learns the long-term dependency relationship of images. However, LBNet only cascades CNN and Transformer, and the local and global features they extract are not well fused. In general, the aforementioned models do not adequately consider the effective aggregation of features extracted by CNN and Transformer, making it challenging to achieve an optimal balance between model size and performance. To strike a compromise between accuracy, complexity, and model size, the network's feature representation must be enhanced within a limited number of parameters. Consequently, we design the CATB, which can adaptively learn the fusion weights between local and global features, thereby improving the network's feature representation.

## 3. Methods

### 3.1. Overview

**Figure 2:**
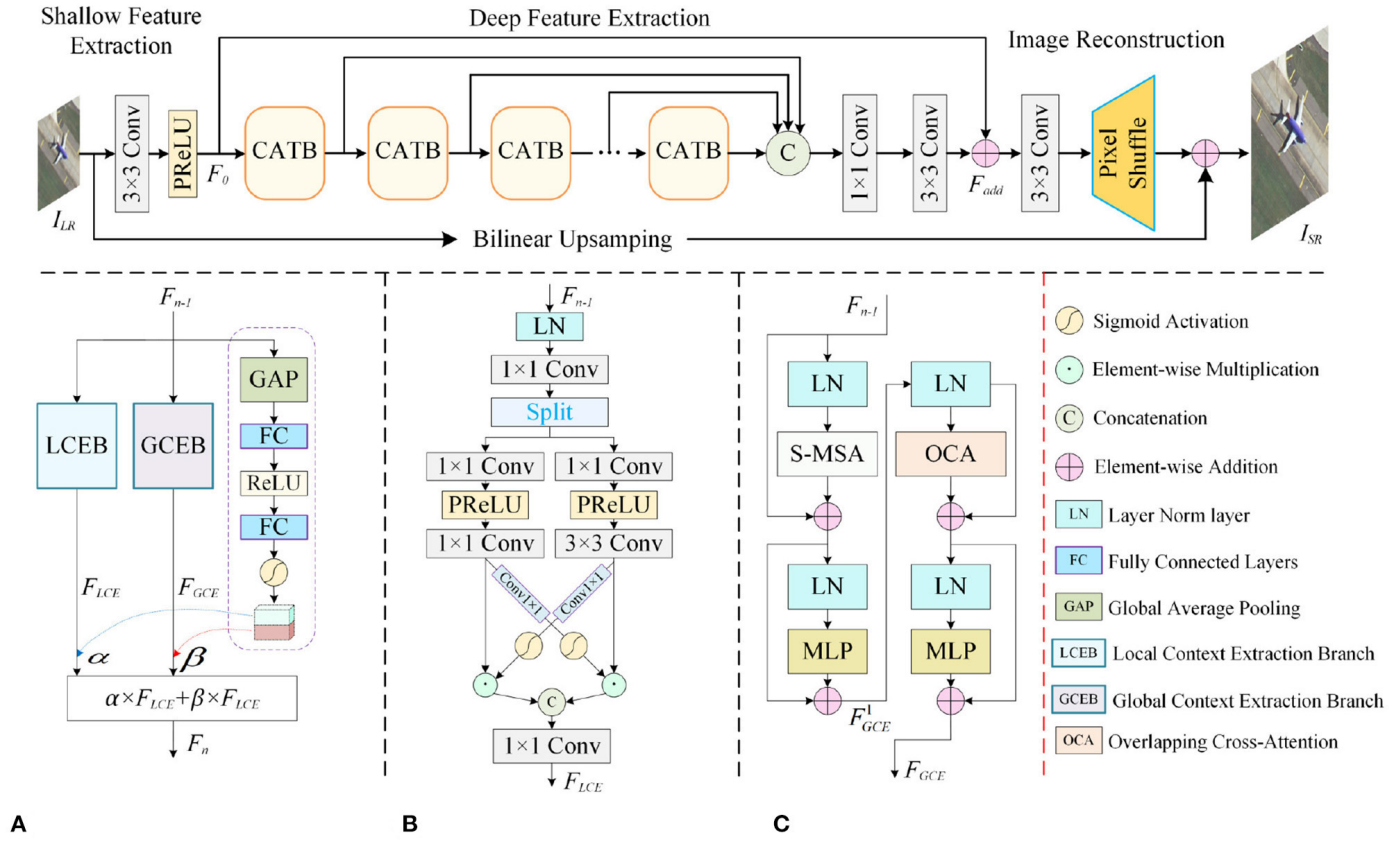
Overall architecture of the proposed CALSRN. **(A)** Context-aware transformer block. **(B)** Local context extraction branch. **(C)** Global context extraction branch.

The proposed context-aware lightweight super-resolution network (CALSRN) consists of three main parts: a shallow feature extraction module, a deep feature extraction module, and a reconstruction layer, as shown in Figure 2. The shallow feature extraction module adopts a 3 × 3 convolution layer and a *PReLU* activation function to extract shallow feature, which contain more fine-grained information. Deep features are extracted through *N* cascaded CATBs, and features from different levels of CATBs are concatenated to obtain SR reconstruction images through reconstruction layer. CATB is a feature extraction block designed based on CNN and Transformer, which is composed of local context extraction branch (LCEB), global context extraction branch (GCEB), and dynamic weight generation branch (DWGB).

### 3.2. Network structure

Given a degraded low-resolution image $I_{LR}\in {\mathbb{R}}^{H\times W\times C_{in}}$, its shallow features $F_{0}\in {\mathbb{R}}^{H\times W\times C}$ are extracted by the shallow feature extraction module, which can be formulated as:


(1) $$\begin{array}{l} F_{0}=PReLU\left(conv_{3\times 3}\left(I_{LR}\right)\right), \end{array}$$

where *C*_*in*_ and *C* denote the number of channels for low-resolution images and its shallow features, respectively. *conv*_3×3_ represents 3 × 3 convolution. *PReLU* is *PReLU* activation function.

***F***_0_ is input to the deep feature extraction module to extract deep features. The deep feature extraction module consists of *N* CATBs. Assuming $F_{n}\in {\mathbb{R}}^{H\times W\times C}$ is the output of the *n*-th CATB, it can be expressed as:


(2) $$\begin{array}{l} F_{n}=f_{CATB}^{n}\left(f_{CATB}^{n-1}\cdot \left(f_{CATB}^{1}\left(F_{0}\right)\right)\right), \end{array}$$

where $f_{CATB}^{n}$ denotes the *n*-th CATB. The outputs of all CATBs are concatenated, and channel downscaling is performed using 1 × 1 convolution, and features of each level are fused by 3 × 3 convolution to obtain deep features. Then, the residual structure is used to sum the deep features and shallow features to obtain the feature $F_{add}\in {\mathbb{R}}^{H\times W\times C}$:


(3) $$\begin{array}{l} F_{add}=conv_{3\times 3}\left(conv_{1\times 1}\left(\left[F_{1},F_{2},\cdot ,F_{N}\right]\right)\right)+F_{0}, \end{array}$$

where [·, ·] denotes concatenation operation. ***F***_*add*_ is fed into the reconstruction layer for super-resolution reconstruction.

The reconstruction layer consists of 3 × 3 convolution and Pixel Shuffle upsampling operation. The reconstructed result of ***F***_*add*_ by the reconstruction layer are summed with the up-sampling result of the low-resolution image to obtain the super-resolution reconstruction image ***I***_*SR*_.


(4) $$\begin{array}{l} I_{SR}=H_{Pi}\left(con_{3\times 3}\left(F_{add}\right)\right)+H_{Bi}\left(I_{LR}\right), \end{array}$$

where *H*_*Pi*_ and *H*_*Bi*_ denote Pixel Shuffle upsampling operation and bilinear upsampling operation, respectively.

The reconstruction loss is used to constrain the proposed network. Assuming that the total number of training samples is *B*, the reconstruction loss can be expressed as:


(5) $$\begin{array}{l} L_{re}=\frac{1}{B}\overset{B}{\underset{i=1}{\sum }}{\left\|I_{SR}^{i}-I_{HR}^{i}\right\|}_{1}, \end{array}$$

where $I_{SR}^{i}$ and $I_{HR}^{i}$ are the *i*-th reconstructed super-resolution image and its corresponding labeled high-resolution image, respectively.

### 3.3. Context-aware transformer block

CATB consists of GCEB, LCEB, and DWGB. GCEB is designed based on the Swin Transformer (Liu et al., 2021) to extract global information. LCEB uses a CNN-based cross-attention mechanism for extracting local information. DWGB adaptively generates fusion weights for weighted fusion of global and local features.

The structure of GCEB is shown in Figure 2C. GCEB can be divided into two layers, which employ S-MSA (Liu et al., 2021) and overlapping cross-attention (Lu et al., 2021) mechanisms to achieve information interaction between windows, respectively. Among them, the first layer uses the S-MSA mechanism, and the window size determines the range of self-attention. A larger window size is beneficial for obtaining more relevant information, but expanding the window size will increase the number of parameters and model complexity. To reduce the computational complexity of the model, we introduce an overlapping cross-attention (OCA) mechanism in the second layer of GCEB. OCA enhances the expression of window self-attention by establishing cross-window connections, which is less computationally demanding than S-MSA. S-MSA is primarily used to capture spatial relationships within the input features. By applying multi-head self-attention on sliding windows, the network can focus on relevant spatial contexts and enhance its perception of local spatial details. On the other hand, OCA effectively aggregates cross-window information while reducing computational complexity, thereby enhancing the interaction between neighboring window features.

Let $F_{n-1}\in {\mathbb{R}}^{H\times W\times C}$ denote the input of the CATB. $F_{n-1}^{1}$ can be obtained after ***F***_*n*−1_ is processed by the first layer of GCEB.


(6) $$\begin{array}{l} F_{n-1}^{1}=MLP\left(LN\left(S-MSA\left(LN\left(F_{n-1}\right)+F_{n-1}\right)\right)\right)\\ \;+\left(S-MSA\left(LN\left(F_{n-1}\right)\right)+F_{n-1}\right), \end{array}$$

where *S*−*MSA* indicates sliding window multi-headed self-attentive operation. *LN* and *MLP* denote layer normalization and multi-layer perceptron, respectively. The output features of the second layer of GCEB are expressed as:


(7) $$\begin{array}{l} F_{GCE}=MLP\left(LN\left(OCA\left(LN\left(F_{n-1}^{1}\right)+F_{n-1}^{1}\right)\right)\right)\\ \;+\left(OCA\left(LN\left(F_{n-1}^{1}\right)\right)+F_{n-1}^{1}\right), \end{array}$$

where ***F***_*GCE*_ is the global feature extracted by GCEB. *OCA* indicates overlapping cross-attention operation.

As shown in Figure 2B, in LCEB, the input feature ***F***_*n*−1_ passes through the LN layer, 1 × 1 convolution for further feature extraction. The extracted features are divided into two parts along the channel: $X\in {\mathbb{R}}^{H\times W\times \frac{C}{2}}$ and $Y\in {\mathbb{R}}^{H\times W\times \frac{C}{2}}$, which can be represented as:


(8) $$\begin{array}{l} \left\{X,Y\right\}=split\left(conv_{1\times 1}\left(LN\left(F_{n-1}\right)\right)\right), \end{array}$$

where *split* is the feature separation operation along the channel. ***X*** and ***Y*** go through 1 × 1 convolution to reshape their channel dimensions to *C*, respectively, and then they are passed through the PReLU activation function to obtain $\tilde{X}$ and $\tilde{Y}$. Convolution operations are performed on $\tilde{X}$ and $\tilde{Y}$ using convolution kernels of different sizes to obtain features ***X***_1_ and ***Y***_1_ with different receptive fields. In order to integrate the features ***X***_1_ and ***Y***_1_, we introduce a cross-attention mechanism. The features obtained by cross-attention fusion are the local features extracted by the network, which can be expressed as:


(9) $$\begin{array}{l} F_{LCE}=conv_{1\times 1}\left[\sigma \left(conv_{1\times 1}\left(X_{1}\right)\right)⊙Y_{1},\sigma \left(conv_{1\times 1}\left(Y_{1}\right)\right)⊙X_{1}\right], \end{array}$$

where σ denotes Sigmoid activation function. ⊙ denotes element-by-element multiplication.

In order to adaptively adjust the fusion weights of global and local features, we introduce a dynamic weight generation branch (DWGB), as shown in Figure 2A. DWGB can adaptively learn the weighted fusion coefficients of global features and local features. The input of DWGB is ***F***_*n*−1_ and the output is a two-dimensional vector [α, β].


(10) $$\begin{array}{l} \left\{\alpha ,\beta \right\}=\sigma \left(FC\left(\gamma \left(GAP\left(F_{n-1}\right)\right)\right)\right), \end{array}$$

where α and β are the fusion weights of local features and global features. γ denotes ReLU activation function. *FC* is fully connected layer. *GAP* denotes global average pooling.

Finally, the output of CTAB is obtained by weighted fusion of local features and global features.


(11) $$\begin{array}{l} F_{n}=\alpha \times F_{LCE}+\beta \times F_{GCE}, \end{array}$$

where ***F***_*n*_ denotes the output of CATB.

## 4. Experimental results and analysis

### 4.1. Experimental setup

The DIV2K dataset (Timofte et al., 2017) was used to train the proposed network. This dataset consists of 800 training images, 100 validation images, and 100 test images, each with 2 K resolution. To comprehensively evaluate the model performance, we used two remote-sensing image datasets RS-T1 and RS-T2 (Wang et al., 2022), as well as five super-resolution benchmark test sets: Set5 (Bevilacqua et al., 2012), Set14 (Zeyde et al., 2012), BSD100 (Huang et al., 2015), Urban100 (Martin et al., 2001), and Manga109 (Matsui et al., 2017) to test the models. PSNR and SSIM (Wang et al., 2004) were used as evaluation metrics to measure the quality of the reconstruction images. PSNR and SSIM are calculated on the Y channel after converting the reconstructed image from RGB space to YCbCR space. In addition, we used the number of parameters (Params) and the number of multiplication and addition operations (Muti-Adds) to evaluate the size and complexity of the model.

In the experiments, low-resolution images were generated from high-resolution images by bicubic downsampling with scale factors of 2×, 3×, and 4×. Moreover, we performed data expansion using random rotations of 90, 180, 270^*o*^ and horizontal flips. The Adam optimizer was used to optimize the proposed network, where β_1_ = 0.9, β_1_ = 0.999, ϵ = 10^−8^. The size of mini-batch was set to 16. The initial learning rate was set to 5 × 10^−4^ and the learning rate was halved every 200 epochs. The total training epoch was 1, 000. The 2× super-resolution model was trained from scratch and it was used as a pre-training model for the 3× and 4× super-resolution models. The number of CATBs in the proposed network was 4, and 50 feature channels were used in the middle layer to ensure the lightweight of the model. All experiments were performed under Pytorch 1.12.1 framework using two NVIDIA GTX3090 GPUs (24 G).

### 4.2. Experiments on remote-sensing image datasets

To validate the effectiveness of the proposed model in this paper, we compare the proposed method with state-of-the-arts methods [SRCNN (Dong et al., 2014), VDSR (Kim et al., 2016a), LGCNet (Lei et al., 2017), LapSRN (Lai et al., 2017), CARN-M (Ahn et al., 2018), IDN (Hui et al., 2018), LESRCNN (Tian et al., 2020), FeNet (Wang et al., 2022), LBNet (Gao et al., 2022b)] on the remote-sensing image datasets RS-T1 and RS-T2 (Wang et al., 2022). Both RS-T1 and RS-T2 consist of 120 images covering 21 complex ground truth remote-sensing scenarios. For a fair comparison, all comparison methods are tested on the RS-T1 and RS-T2 datasets using models trained on the DIV2K dataset. Tables 1–3 demonstrate the results of the quantitative evaluation of the compared methods on the RS-T1 and RS-T2 datasets. According to Tables 1–3 that the PSNR/SSIM values of the 2×, 3×, 4× super-resolution reconstruction results of the proposed method on RS-T1 and RS-T2 datasets are optimal. Moreover, the Multi-Adds value of the proposed method is the best, and the number of parameters is about 30 K less than that of FeNet, which is the current optimal lightweight super-resolution reconstruction model for remote-sensing images. It confirms that the proposed method can achieve good performance with a small number of parameters.

**Table 1:** Quantitative comparison of 2× super-resolution results obtained by different methods on RS-T1 and RS-T2 datasets.

**Method**	**Params (K)**	**Multi-adds (G)**	**PSNR/SSIM**	
			**RS-T1**	**RS-T2**
SRCNN	57	52.7	35.18/0.9243	32.87/0.9209
VDSR	666	612.6	35.85/0.9312	33.86/0.9312
LGCNet	193	178.1	35.65/0.9298	33.47/0.9281
LapSRN	251	29.9	35.69/0.9304	33.57/0.9286
CARN-M	412	91.2	35.77/0.9314	33.84/0.9315
IDN	553	124.6	36.13/0.9339	34.07/0.9329
LESRCNN	626	281.5	36.04/0.9328	34.00/0.9320
FeNet	351	77.9	36.23/0.9341	34.22/0.9337
LBNet	731	153.2	36.28/0.9345	34.30/0.9339
Proposed	319	20.4	**36.34**/**0.9356**	**34.37**/**0.9349**

Multi-Adds is computed according to a 1, 280 × 720 image.

The bold values represent the best performance.

**Table 2:** Quantitative comparison of 3× super-resolution results obtained by different methods on RS-T1 and RS-T2 datasets.

**Method**	**Params (K)**	**Multi-Adds (G)**	**PSNR/SSIM**	
			**RS-T1**	**RS-T2**
SRCNN	57	52.7	30.95/0.8228	28.59/0.8180
VDSR	666	612.6	31.55/0.9352	29.40/0.8391
LGCNet	193	79.0	31.30/0.8314	29.03/0.8312
LapSRN	290	115.2	31.47/0.8338	29.22/0.8352
CARN-M	412	46.1	31.72/0.8426	29.62/0.8452
IDN	553	56.3	31.73/0.8430	29.59/0.8450
LESRCNN	810	238.9	31.68/0.8398	29.65/0.8444
FeNet	357	35.2	31.89/0.8432	29.80/0.8481
LBNet	736	51.5	31.96/0.8485	29.91/0.8516
Proposed	326	20.8	**32.05**/**0.8505**	**30.01**/**0.8526**

Multi-Adds is computed according to a 1, 280 × 720 image.

The bold values represent the best performance.

**Table 3:** Quantitative comparison of 4× super-resolution results obtained by different methods on RS-T1 and RS-T2 datasets.

**Method**	**Params (K)**	**Multi-Adds (G)**	**PSNR/SSIM**	
			**RS-T1**	**RS-T2**
SRCNN	57	52.7	28.87/0.7382	26.46/0.7296
VDSR	666	612.6	29.33/0.7546	27.03/0.7525
LGCNet	193	44.5	29.13/0.7481	26.76/0.7426
LapSRN	543	139.6	29.51/0.7614	27.24/0.7600
CARN-M	412	32.5	29.57/0.7624	27.37/0.7647
IDN	553	32.3	29.56/0.7623	27.31/0.7627
LESRCNN	774	241.6	29.62/0.7625	27.41/0.7646
FeNet	366	20.4	29.70/0.7688	27.45/0.7672
LBNet	742	38.9	29.78/0.7689	27.52/0.7732
Proposed	336	21.4	**29.85**/**0.7717**	**27.67**/**0.7759**

Multi-Adds is computed according to a 1, 280 × 720 image.

The bold values represent the best performance.

**Figure 3:**
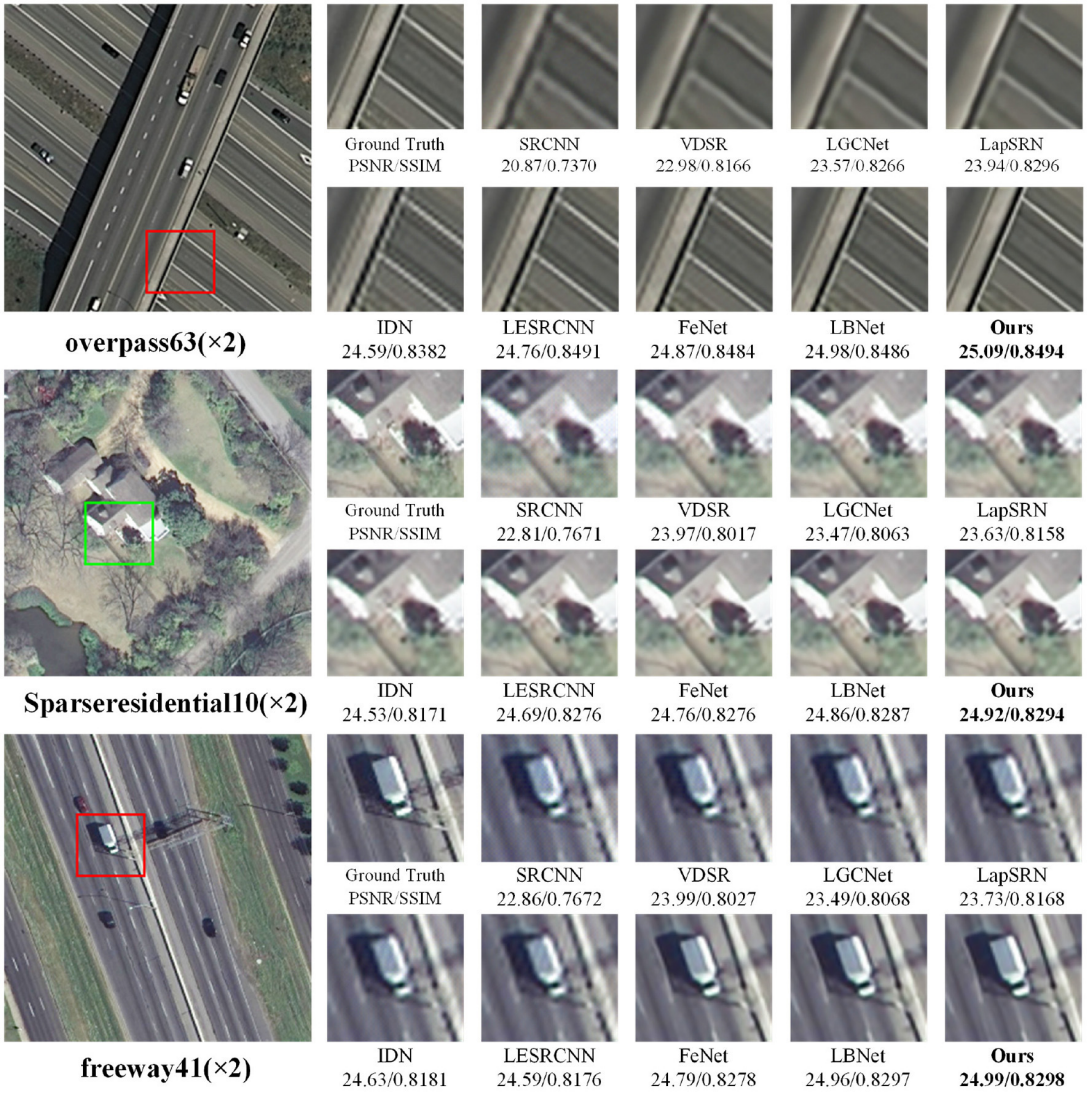
Visual comparisons with different methods for 2× super-resolution on RS-T1 and RS-T2 datasets.

**Figure 4:**
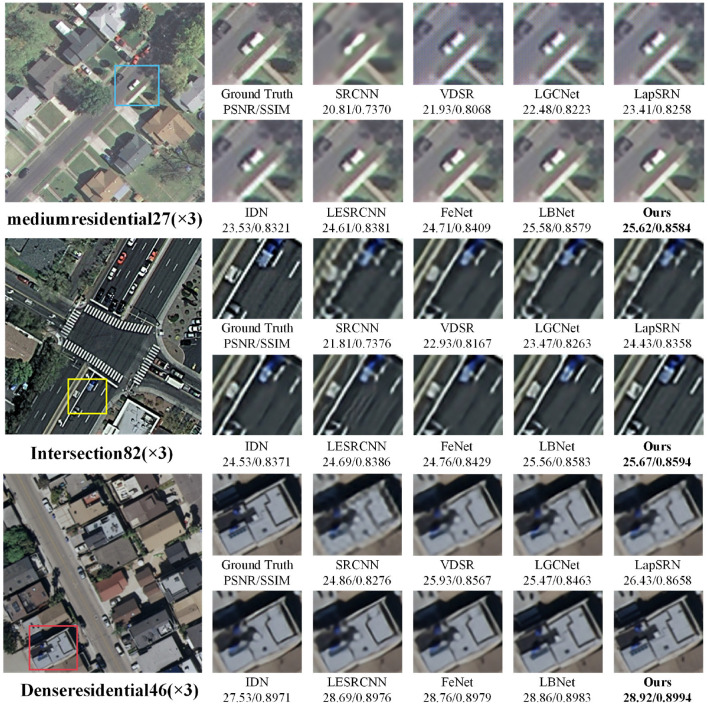
Visual comparisons with different methods for 3× super-resolution on RS-T1 and RS-T2 datasets.

**Figure 5:**
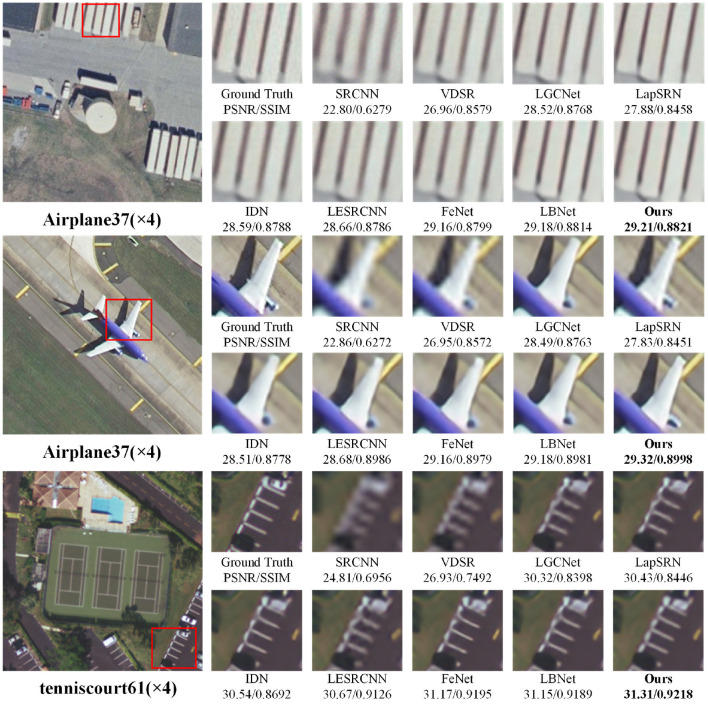
Visual comparisons with different methods for 4× super-resolution on RS-T1 and RS-T2 datasets.

In addition, the 2×, 3×, and 4× super-resolution reconstruction results of remote-sensing images are illustrated in Figures 3–5, respectively. As shown in Figure 3, when the magnification factor is 2, the visual effect of the proposed method on “overpass63,” “Sparseresidential10,” and “freeway41” is better than that of the comparison methods in terms of clarity, and the PSNR and SSIM values are also optimal. As shown in Figure 4, the 3× reconstructed images of the proposed method achieve the optimal quality in terms of both structure and detailed texture, especially for the “Denseresidential46” image, where the comparison methods fail to recover the corner information. As shown in Figure 5, IDN, FeNet, LBNet, and our proposed method all achieve good visual results, while the reconstructed images of the remaining comparison methods are relatively blurry. Overall, as a lightweight super-resolution model, the proposed model achieves better quantitative and qualitative results than existing models.

### 4.3. Experiments on super-resolution benchmark test sets

To further verify the generalization of the proposed model in this paper, we conduct comparison experiments on the benchmark test sets. The datasets Set5, Set14, BSD100, Urban100, and Manga109 are benchmark test sets for image super-resolution reconstruction, covering images of different scenes such as urban buildings, animals, plants, and animations. In order to verify the effectiveness of the proposed method, we compare the proposed method with state-of-the-arts methods [SRCNN (Dong et al., 2014), FSRCNN (Dong et al., 2016), LapSRN (Lai et al., 2017), VDSR (Kim et al., 2016a), LGCNet (Lei et al., 2017), DRRN (Tai et al., 2017a), CARN-M (Ahn et al., 2018), IDN (Hui et al., 2018), MemNet (Tai et al., 2017b), LESRCNN (Tian et al., 2020), MADNet (Lan et al., 2021), FDIWN (Gao et al., 2022a), LBNet (Gao et al., 2022b)] on the five test sets mentioned above. It is worth noting that the models for the comparison methods are the already trained models provided by the original authors. The objective evaluation results of the 3× and 4× magnification factor super-resolution reconstruction experiments are shown in Tables 4, 5. The best values are highlighted in bold. As shown in Tables 4, 5, the PSNR/SSIM values of the proposed method outperforms the others at the most metrics. Moreover, compared with the LBNet and FDIWN methods, which have comparable performance to the proposed method, they have more than twice the number of parameters and much larger Multi-Adds than those of the proposed method. Overall, the proposed model achieves a good balance among the number of parameters, complexity and performance.

**Table 4:** Quantitative comparison of 3× super-resolution results obtained by different methods on super-resolution benchmark datasets.

**Method**	**Params (K)**	**Multi-Adds (G)**	**PSNR/SSIM**				
			**Set5**	**Set14**	**BSD100**	**Urban100**	**Manga109**
SRCNN	57	52.7	32.75/0.9090	29.30/0.8215	28.41/0.7863	26.24/0.7989	30.48/0.9117
FSRCNN	54	5.0	33.18/0.9140	29.37/0.8240	28.53/0.7910	26.43/0.8080	31.10/0.9210
LapSRN	502	115.2	33.81/0.9220	29.79/0.8325	28.82/0.7980	27.07/0.8275	32.21/0.9350
VDSR	666	612.6	33.66/0.9213	29.77/0.8314	28.82/0.7976	27.14/0.8279	32.01/0.9340
LGCNet	193	79.0	33.32/0.9172	29.67/0.8289	28.63/0.7923	26.77/0.8180	–
DRRN	298	6796,9	34.03/0.9244	29.96/0.8349	28.95/0.8004	27.53/0.8378	32.71/0.9379
CARN-M	412	46.1	33.99/0.9236	30.08/0.8367	28.91/0.8000	27.55/0.8385	32.78/0.9384
IDN	553	56.3	34.11/0.9253	29.99/0.8354	28.95/0.8013	27.42/0.8359	32.71/0.9381
MemNet	678	2662.4	34.09/0.9248	30.00/0.8350	28.96/0.8001	27.56/0.8376	32.51/0.9369
LESRCNN	810	238.9	33.93/0.9231	30.12/0.8380	28.91/0.8005	27.70/0.8415	32.76/0.9389
MADNet	930	88.4	34.14/0.9251	30.20/0.8395	28.98/0.8023	27.78/0.8439	–
LBNet	736	68.4	34.47/0.9277	30.38/0.8417	29.13/0.8061	28.42/0.8559	33.80/0.9430
FDIWN	645	51.5	**34.52**/0.9281	30.42/0.8438	29.14/0.8065	28.35/0.8567	–
Proposed	326	20.8	34.50/**0.9283**	**30.52**/**0.8455**	**29.17**/**0.8085**	**28.49**/**0.8586**	**33.99**/**0.9470**

−Indicates that the result is unknown. Multi-Adds is computed according to a 1, 280 × 720 image.

The bold values represent the best performance.

**Table 5:** Quantitative comparison of 4× super-resolution results obtained by different methods on super-resolution benchmark datasets.

**Method**	**Params (K)**	**Multi-Adds (G)**	**PSNR/SSIM**				
			**Set5**	**Set14**	**BSD100**	**Urban100**	**Manga109**
SRCNN	57	52.7	30.48/0.8626	27.50/0.7513	26.90/0.7101	24.52/0.7221	27.58/0.8555
FSRCNN	54	4.6	30.72/0.8660	26.98/0.7150	26.98/0.7150	24.62/0.7280	27.90/0.8610
LapSRN	543	139.6	31.54/0.8852	28.09/0.7700	27.32/0.7275	25.21/0.7562	29.09/0.8900
VDSR	666	612.6	31.35/0.8838	28.01/0.7674	27.29/0.7251	25.18/0.7524	28.83/0.8870
LGCNet	193	44.5	30.87/0.8746	27.82/0.7630	27.08/0.7186	24.82/0.7399	-
DRRN	298	6796.9	31.68/0.8888	28.21/0.7720	27.38/0.7284	25.44/0.7638	29.45/0.8946
CARN-M	412	32.5	31.92/0.8903	28.42/0.7762	27.44/0.7304	25.63/0.7688	29.80/0.8989
IDN	553	32.3	31.82/0.8903	28.25/0.7730	27.41/0.7297	25.41/0.7632	29.41/0.8942
MemNet	678	2662.4	31.74/0.8893	28.26/0.7723	27.40/0.7281	25.50/0.7630	29.42/0.8942
LESRCNN	774	241.6	31.88/0.8903	28.44/0.7772	27.45/0.7313	25.77/0.7732	29.94/0.9002
MADNet	1002	54.1	32.01/0.8925	28.45/0.7781	27.47/0.7327	25.77/0.7751	–
LBNet	742	38.9	**32.29**/0.8960	28.68/0.7832	27.62/0.7382	26.27/0.7906	30.76/0.9111
FDIWN	664	28.4	32.23/08955	28.66/07829	27.62/07380	26.28/07919	–
Proposed	336	21.4	32.27/**0.8965**	**28.70**/**0.7845**	**27.66**/**0.7409**	**26.53**/**0.7986**	**30.98**/**0.9145**

−Indicates that the result is unknown. Multi-Adds is computed according to a 1, 280 × 720 image.

The bold values represent the best performance.

**Figure 6:**
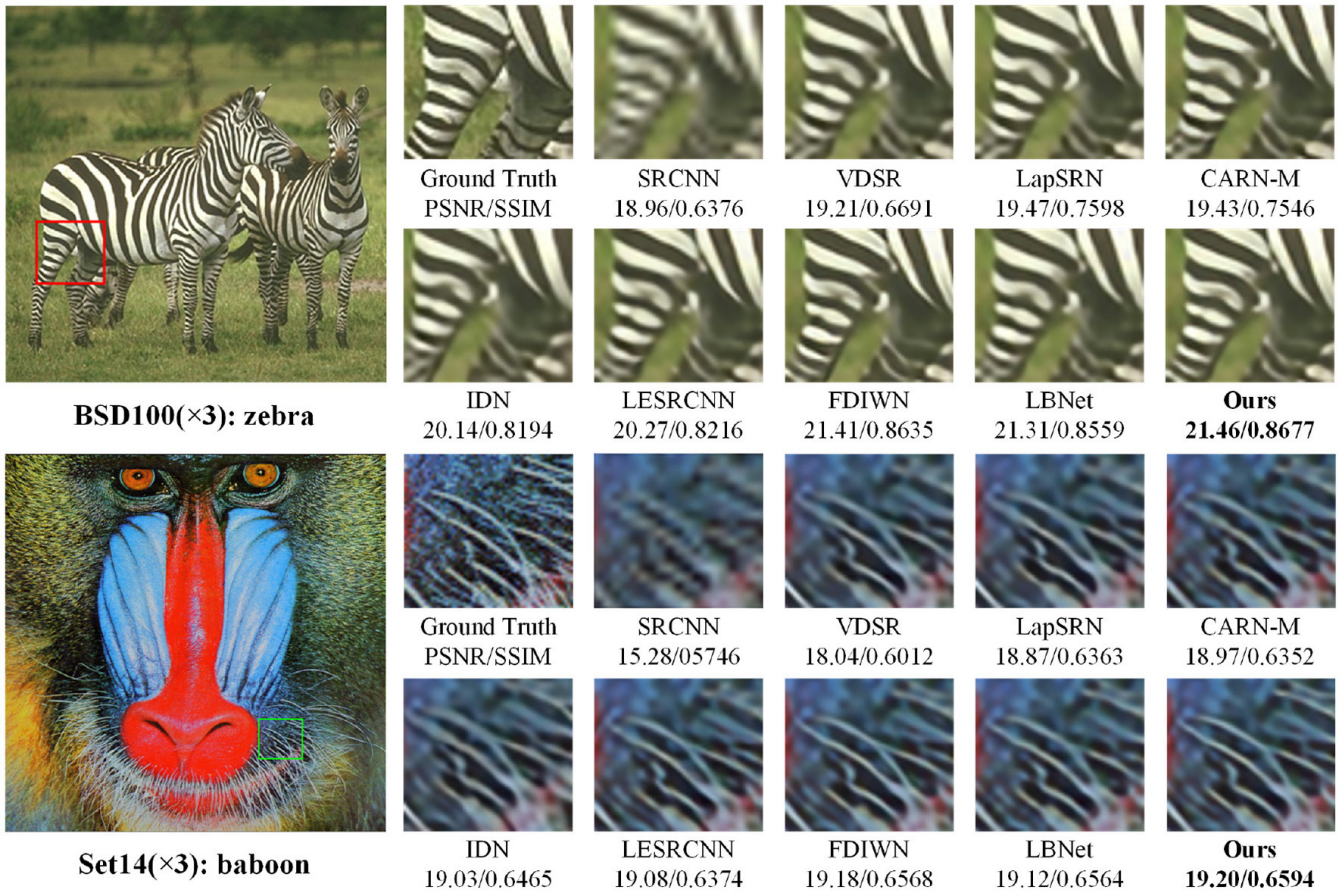
Visual comparisons with different methods for 3× super-resolution on super-resolution benchmark datasets.

**Figure 7:**
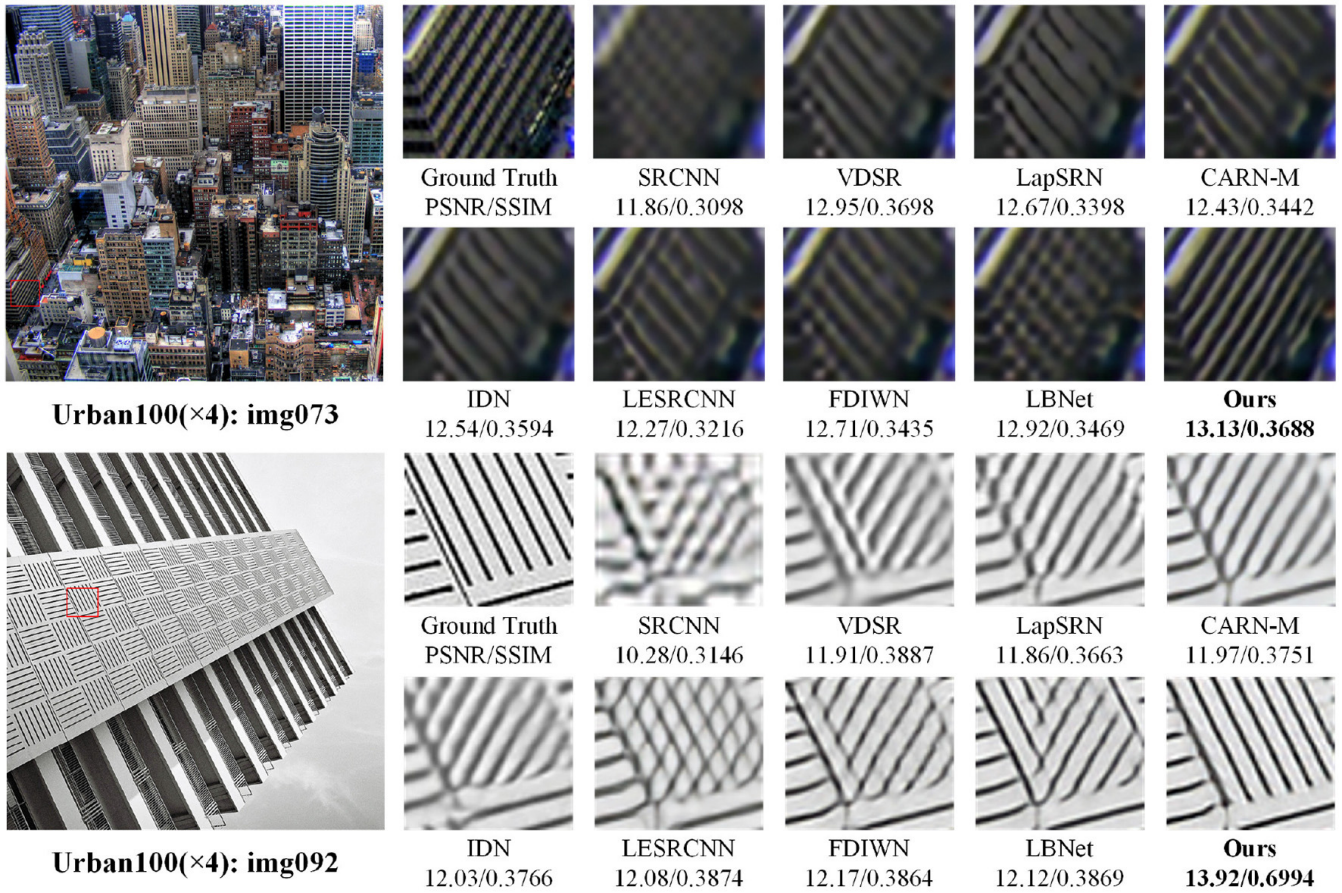
Visual comparisons with different methods for 4× super-resolution on super-resolution benchmark datasets.

To evaluate the visual quality of the super-resolution reconstruction images, the 3× and 4× super-resolution images are shown in Figures 6, 7, respectively. As shown in Figure 6, when the images are enlarged by 3 times, artifacts are introduced in the reconstruction results of the comparison methods. As shown in Figure 7, the 4× super-resolution results of the “img073” and “img092” images in the Urban test set are closest to the Ground-Truth images, and achieve the best visual experience in terms of overall image clarity and detail texture. Other comparison methods exhibit visible artifacts, such as severe misalignment in the locally zoomed-in “img092” images restored by SRCNN, VDSR, LapSRN, CARN-M, IDN, LESRCNN, and FDIWM. Overall, compared with existing methods, the visual quality of the reconstructed images by the proposed method is optimal in terms of clarity and detailed texture.

### 4.4. Ablation study

To verify the effectiveness of the global context extraction branch (GCEB), local context extraction branch (LCEB) and dynamic weight generation branch (DWGB) proposed in this work, we conduct ablation experiments on the RS-T1 dataset with a magnification factor of 2. The results of the ablation experiments are shown in Table 6. In the ablation experiment, this paper removes DWGB, GCEB, and LCEB one by one from the complete model, and then compare the performance of the modified model with the complete model. As shown in Table 6, the model performance all decreases when DWGB, GCEB, and LCEB are removed from the complete model. This indicates that DWGB, GCEB, and LCEB all have a positive effect on improving the model performance.

**Table 6:** Ablation study of each module on the RS-T1 dataset with a magnification factor of 2.

**LCEB**	**GCEB**	**DWGB**	**Params (K)**	**Multi-Adds (G)**	**PSNR/SSIM**
✓	✓	✓	319	20.4	36.29/0.9343
✓	✓	×	304	15.9	36.11/0.9332
✓	×	×	156	15.1	36.02/0.9328
×	✓	×	256	15.3	36.06/0.9329

**Figure 8:**
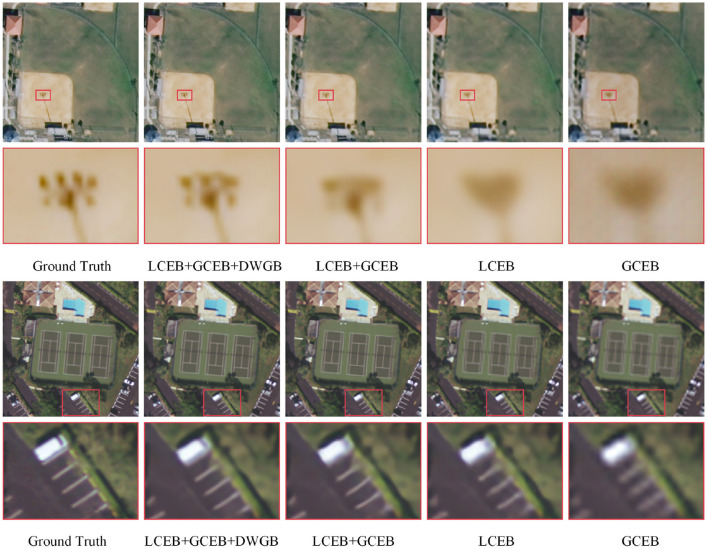
Visual results of 2× ablation experiments on remote sensing test set RS-T1.

We use the “denseresidential13” and “baseballdiamond98” images from the RS-T1 test set to verify the ablation experiment visually. From the local zoom-in visual results in Figure 8, it can be seen that the image quality decreases when DWGB, GCEB and LCEB are removed from the complete model one by one. The combination of LCEB+GCEB+DWGB achieves the best visual performance, and when DWGB is removed from the complete model, the model's performance decreases, resulting in blurry images for “denseresidential13” and “baseballdiamond98.” This demonstrates the crucial role of the dynamic weight generation branch (DWGB) in adjusting global and local information within the overall network. When the model only has LCEB or GCEB, the reconstructed images is blurry. The visual results in Figure 8 confirm the effectiveness of the proposed modules. LCEB captures local details, GCEB extracts global information, and DWGB dynamically assigns weights and fuse the local and global features.

In addition, we analyze the effect of the number of CATBs on the performance of the proposed model. The experiment is conducted on the RS-T1 dataset with a magnification factor of 2. The results of the experiment are shown in Table 7. As shown in Table 7, the PSNR/SSIM values of the reconstructed images improve as the number of CATB blocks increases, but the number of parameters and the complexity of the model also increase. When the number of CATB blocks is 3, the model has the smallest number of parameters and computational complexity, but the PSNR and SSIM of the reconstructed images are also the lowest. When the number of CATB blocks is increased to 6, the best performance is achieved, but the number of model parameters and computational complexity are too large. Therefore, to balance the number of parameters and the performance of the model, we set the number of CATB blocks to 4.

**Table 7:** Performance of the proposed model on RS-T1 with different number of CATBs.

**CATBs**	**Params (K)**	**Multi-Adds (G)**	**PSNR/SSIM**
3	245.7	16.8	36.18/0.9322
4	319.8	20.4	36.30/0.9343
5	406.2	26.8	36.38/0.9347
6	478.3	30.7	36.42/0.9350

## 5. Conclusion

We propose a lightweight SISR network called CLASRN for the super-resolution reconstruction of low-resolution remote-sensing images. CLASRN combines the advantages of Transformer and CNN to better recover local details while emphasizing long-range information in images. Furthermore, the proposed network dynamically adjusts fusion weights between local and global features to enhance the network's feature extraction capability. Compared with other methods, the proposed method reconstructs high-quality images with a smaller number of parameters and lower computational complexity. Through the analysis of visual results, we have found that the proposed method has advantages over other comparison methods in restoring local details and global information of the image. Finally, experimental results on two remote-sensing datasets and five SR benchmark datasets demonstrate that our network can better achieve a balance between performance and model complexity.

To address the challenge of large model parameters that can hinder model deployment, we have developed a lightweight super-resolution reconstruction network that reduces computational complexity and model size while ensuring high-quality image reconstruction. In the future, we intend to investigate practical deployment techniques for lightweight super-resolution models, making them more compatible with lower-performance hardware devices, such as embedded and mobile devices. Additionally, there is still room for improvement in our model, particularly in real application scenarios. To improve the restoration quality of low-resolution images in real-world scenarios, we plan to explore the integration of blind super-resolution methods with supervised end-to-end training, aiming to design a model that can reconstruct super-resolution images in real-world situations.

## Data availability statement

The original contributions presented in the study are included in the article/supplementary material, further inquiries can be directed to the corresponding author.

## Author contributions

GP was responsible for paper scheme design, experiment, and paper writing. MX guided the paper scheme design, experiments, and wrote the papers. LF guided paper writing, revision, translation, and typesetting. All authors contributed to the article and approved the submitted version.
